# *SPAG1* Expression as a Candidate Predictor of Pathological Lymph Node Metastasis in Prostate Cancer: A Transcriptomic Analysis of The Cancer Genome Atlas Prostate Adenocarcinoma Cohort

**DOI:** 10.3390/genes17080875

**Published:** 2026-07-28

**Authors:** Ebtihal Alharbi, Yousef Almehmadi

**Affiliations:** 1 Department of Pathology, Faculty of Medicine, King Abdulaziz University, Rabigh 21589, Saudi Arabia; 2 Department of Surgery, Faculty of Medicine, King Abdulaziz University, Rabigh 21589, Saudi Arabia; yalmehmadi@kau.edu.sa

**Keywords:** prostate cancer, lymph node metastasis, biomarker, RNA sequencing, gene set enrichment analysis, logistic regression, TCGA

## Abstract

**Background/Objectives:** Improved preoperative prediction of nodal metastasis in prostate cancer could refine selection for extended pelvic lymph node dissection, a high-morbidity procedure. Sperm-associated antigen 1 (*SPAG1*) is a candidate marker of nodal status, but its incremental value beyond clinical staging and the associated transcriptional state remain unevaluated. **Methods:** In The Cancer Genome Atlas prostate adenocarcinoma (TCGA-PRAD) cohort (497 patients with matched clinical and RNA-sequencing data), we evaluated the association between *SPAG1* expression and pathological N stage by logistic regression with 2000-resample bootstrap optimism correction and sensitivity analyses for missing nodal data and batch effects. Hallmark enrichment analysis compared *SPAG1* expression extremes (quartile 4 vs. 1) and, separately, N1 versus N0 tumours adjusted for T stage, Gleason grade, and tissue source site; directional concordance was assessed. **Results:** N1 rates rose across *SPAG1* quartiles from 7.6% to 39.0% (per-quartile odds ratio [OR], 1.83; *p* = 2.55 × 10^−5^). After adjusting for T stage and Gleason grade, *SPAG1* remained an independent predictor (adjusted OR, 2.14; 95% confidence interval [CI], 1.50–3.13; *p* = 4.8 × 10^−5^), stable across both sensitivity analyses. Adding *SPAG1* improved discrimination (area under the receiver-operating characteristic curve, 0.783 to 0.838; ΔAUC, 0.056; paired DeLong *p* = 3.03 × 10^−5^). The *SPAG1* transcriptional programme showed cell-cycle, immune–inflammatory, and mTORC1/TGF-β signalling activation with suppressed differentiation and metabolism; all 15 overlapping Hallmark pathways were directionally concordant with the adjusted N1 signature. **Conclusions:** *SPAG1* expression in primary prostate tumours is a candidate predictor of pathological lymph node metastasis with statistically robust incremental discrimination beyond clinical staging. Independent external validation and biopsy-based feasibility studies are required before clinical application.

## 1. Introduction

Pathological lymph node involvement at radical prostatectomy is among the strongest adverse prognostic features in prostate cancer and a key determinant of postoperative management [1]. Patients with pN1 disease are at increased risk for progression and adverse oncological outcomes; therefore, adjuvant systemic therapy, radiotherapy, and clinical trial enrolment are considered [2]. Identification of nodal involvement relies on extended pelvic lymph node dissection at the time of radical prostatectomy; however, this procedure is associated with substantial morbidity that could improve with enhanced patient selection [2]. Primary tumour transcriptional features can capture biological aspects of metastatic potential that are not fully reflected in conventional pathological variables such as the T stage and Gleason grade, thus raising the possibility that molecular markers derived from tumour tissue could complement pathological staging when identifying patients at risk for nodal spread [3].

Sperm-associated antigen 1 (SPAG1) is a testis-selective protein involved in spermatogenesis that is classified as a candidate cancer-testis antigen because of its tumour-associated expression and immunogenicity [4]. Beyond its reproductive role, SPAG1 is a multidomain tetratricopeptide-repeat-containing protein implicated in protein-complex assembly and the cytoplasmic preassembly of axonemal dyneins [5]. Experimental studies have shown that SPAG1 associates with tubulin and that its knockdown reduces tumour-cell motility in pancreatic cancer cells, as well as proliferative capacity and MEK/ERK phosphorylation in AML cells, although its cancer-specific molecular mechanisms remain incompletely understood [6,7]. Across pancreatic, breast, and haematological malignancies, *SPAG1* overexpression has been associated with tumour progression and adverse outcomes, including lymph node involvement in pancreatic cancer and impaired relapse-free survival in breast cancer, and functional studies have supported its role in tumour-cell motility [6,8,9]. A machine-learning analysis of The Cancer Genome Atlas prostate adenocarcinoma (TCGA-PRAD) integrating RNA expression and copy number alteration data identified *SPAG1* as a candidate gene that discriminates N1 disease from N0 disease (area under the receiver-operating characteristic [ROC] curve [AUC], 0.72, the highest among identified candidates) and found that *SPAG1* copy number amplification is associated with reduced survival [10].

Despite these findings, key questions about the association between *SPAG1* and nodal metastasis are unresolved. Whether *SPAG1* adds discriminative value for the nodal status beyond the T stage and Gleason grade has not been determined. Furthermore, whether the association is robust to institutional batch effects has not been assessed. Additionally, the transcriptional programmes co-expressed with *SPAG1* in node-positive disease have not been characterised.

Therefore, we performed a focused analysis of *SPAG1* in TCGA-PRAD to achieve the following three objectives: quantify the association between *SPAG1* expression and pathological lymph node metastasis after adjusting for the T stage and Gleason grade with sensitivity analyses that address missing nodal data and technical batch effects; evaluate the incremental discriminative value of *SPAG1* beyond a clinical baseline model using paired AUC comparisons and bootstrap internal validation; and characterise the *SPAG1*-associated transcriptional programme and assess its concordance with the molecular signature of node-positive disease. Because this was a discovery and prioritisation study, external validation and experimental validation were not performed; however, they are required to establish clinical applicability.

## 2. Materials and Methods

### 2.1. Cohort and Data Sources

Clinical, survival, and RNA-sequencing (RNA-seq) data for the TCGA prostate adenocarcinoma cohort were obtained from the UCSC Xena platform [11,12]. Survival endpoints were based on the harmonised TCGA Pan-Cancer Clinical Data Resource [13]. The analysis cohort comprised 497 patients with matched clinical, survival, and log2-transformed RSEM-normalised RNA-seq data (Illumina HiSeq V2). All patients were male, as prostate cancer is a sex-specific malignancy; therefore, sex-stratified analyses were not applicable. The progression-free interval (PFI) was selected as the primary survival endpoint based on the event count and follow-up completeness.

### 2.2. Feature Engineering

The pathological T stage was dichotomised as T2 and T3/T4, and the pathological N stage was dichotomised as N0 and N1. Patients who lacked a recorded pathological N stage (*n* = 73; hereafter referred to as patients missing N) were treated as missing in primary analyses. The Gleason score was dichotomised as ≤7 and ≥8. *SPAG1* expression was extracted from the log2 RSEM matrix.

### 2.3. Statistical Analysis Overview

The following three analyses were performed: a logistic regression analysis that evaluated whether *SPAG1* expression predicts the pathological N1 status beyond established clinicopathological variables with internal validation; a parallel Cox survival analysis that extended the signal to disease progression; and a transcriptional analysis that characterised the *SPAG1*-associated gene expression programme and its concordance with the nodal disease signature. Sensitivity analyses addressed potential sources of bias, including indeterminate nodal status and institutional batch effects.

### 2.4. Logistic Regression and Incremental Discrimination

Logistic regression was performed on the complete-case dataset (*n* = 420) to evaluate the association between *SPAG1* expression and the N1 status. A primary model comprising the T stage, Gleason grade, and *SPAG1* was compared against a clinical baseline of T stage and Gleason grade alone using the Akaike information criterion (AIC) and likelihood ratio test; model assumptions including multicollinearity, convergence, separation, and linearity of the continuous predictor were verified, and no major violations were identified. Discrimination was assessed using the AUC, and the incremental value of *SPAG1* over the clinical baseline was evaluated using the paired DeLong test and 2000-resample bootstrap percentile confidence intervals (CIs) for the ΔAUC. Internal validation was performed using 2000-resample bootstrap optimism correction for the AUC and calibration slope, and calibration was assessed using the calibration plot and Hosmer–Lemeshow test.

### 2.5. Sensitivity Analyses

Robustness of the *SPAG1* and N1 association was evaluated under two sensitivity analyses re-fitting the primary logistic model: (i) reclassifying patients with missing N as node-negative (missing data assumption); (ii) additional adjustment for the tissue source site (technical batch).

To assess whether *SPAG1* expression co-varied with bulk immune or stromal cell infiltration (factors known to influence bulk RNA-seq signals) Spearman’s correlation coefficients were computed between *SPAG1* expression and ESTIMATE-derived ImmuneScore and StromalScore [14] across the full cohort. The correlation between *SPAG1* expression and tumour purity in TCGA-PRAD was additionally obtained from TIMER2.0 [15] via the web portal (https://timer.cistrome.org/) using Spearman’s method.

### 2.6. Survival Analysis

Cox proportional hazards models were fitted to evaluate the association between *SPAG1* expression (continuous) and PFI. These models were unadjusted (*n* = 497; 93 events) and adjusted for the pathological T stage, N stage, and Gleason grade (*n* = 420 complete cases; 84 events). The proportional hazards assumption was assessed using Schoenfeld residuals. To enable visualisation, Kaplan–Meier curves were generated using median dichotomisation of *SPAG1*, and curves were displayed up to 10 years. Only four patients had follow-up exceeding this threshold. The median follow-up period was 2.16 years (interquartile range [IQR], 1.16–3.73 years). In a subgroup analysis, the univariable association between *SPAG1* expression (continuous) and PFI was additionally examined separately within the node-negative (N0) and node-positive (N1) strata, with corresponding Kaplan–Meier log-rank tests using median dichotomisation.

### 2.7. Differential Expression and Pathway Analysis

To characterise the *SPAG1*-associated transcriptional programme, patients in the highest and lowest *SPAG1* expression quartiles (quartile 4 [Q4] and quartile 1 [Q1], respectively) were compared using linear modelling with empirical Bayes moderation (limma) [16] and a gene set enrichment analysis (GSEA) of the Hallmark collection using fgsea [17]. To assess whether tissue source site (TSS) adjustment was warranted in the *SPAG1* quartile grouping, TSS distribution was assessed across *SPAG1* expression quartile groups using the chi-square test. No significant association was observed (χ2 = 4.15; *p* = 0.24), indicating that TSS was not a confounder of the *SPAG1* quartile grouping. To define the transcriptional signature of nodal metastasis, parallel differential expression and enrichment analyses that compared N1 and N0 tumours were adjusted for the established clinicopathological correlates of nodal disease (T stage and Gleason grade) and tissue source site as a technical batch covariate. As an additional analysis to assess whether the *SPAG1*-associated programme was preserved across nodal strata, the Hallmark GSEA comparing high versus low *SPAG1* expression was repeated separately within N0 and N1 tumours. Within each stratum, patients were dichotomised at the stratum-specific median *SPAG1* expression rather than by quartile extremes to preserve adequate group sizes, particularly in the smaller N1 subgroup; differential expression (limma) and Hallmark enrichment (fgsea) were otherwise performed as described above, without additional covariate adjustment.

### 2.8. Pathway-Level Concordance

Pathway-level concordance between the two analyses was assessed by intersecting pathways significant at a false discovery rate (FDR) <0.05 in each analysis, with directional agreement evaluated by the sign of normalised enrichment scores. The concordance analysis paired the *SPAG1* Q4 vs. Q1 analysis with the adjusted N1 vs. N0 signature (T stage, Gleason grade, and tissue source site).

### 2.9. Software

All analyses were performed in R version 4.4.3 using the tidyverse packages dplyr (1.2.0) and ggplot2 (4.0.2), survival (3.8-6), limma (3.60.6), fgsea (1.30.0), ESTIMATE (1.0.13), pROC (1.19.0.1), car (3.1-5), msigdbr (26.1.0), and ResourceSelection (0.3-6).

## 3. Results

### 3.1. Cohort Characteristics

A total of 497 patients with matched clinical and RNA-seq data were included. The median follow-up period was 2.16 years (IQR, 1.16–3.73 years; maximum, 13.75 years). Of these patients, 424 had a documented pathological nodal status (N0 or N1) and 420 comprised the complete-case cohort for the primary logistic regression after excluding four patients with missing T stage information. Seventy-nine (18.8%) patients had nodal metastasis. Seventy-three patients had no recorded pathological nodal status and predominantly lower-stage and lower-grade disease than that of patients who underwent surgical nodal evaluations (Table 1; Supplementary Figure S1), thus reflecting selective omission of pelvic lymph node dissection in clinically lower-risk cases. Therefore, these patients were treated as missing in primary analyses, and robustness was confirmed by conducting a sensitivity analysis and reclassifying patients with missing N as node-negative.

*SPAG1* expression was significantly higher in tumours with adverse pathological features (N1, T3/T4, Gleason score ≥ 8), thus providing initial evidence of an association with the disease extent (Table 1; Supplementary Figure S2).

### 3.2. Association Between SPAG1 and the PFI Captured by the Clinical Stage

**Figure 1:**
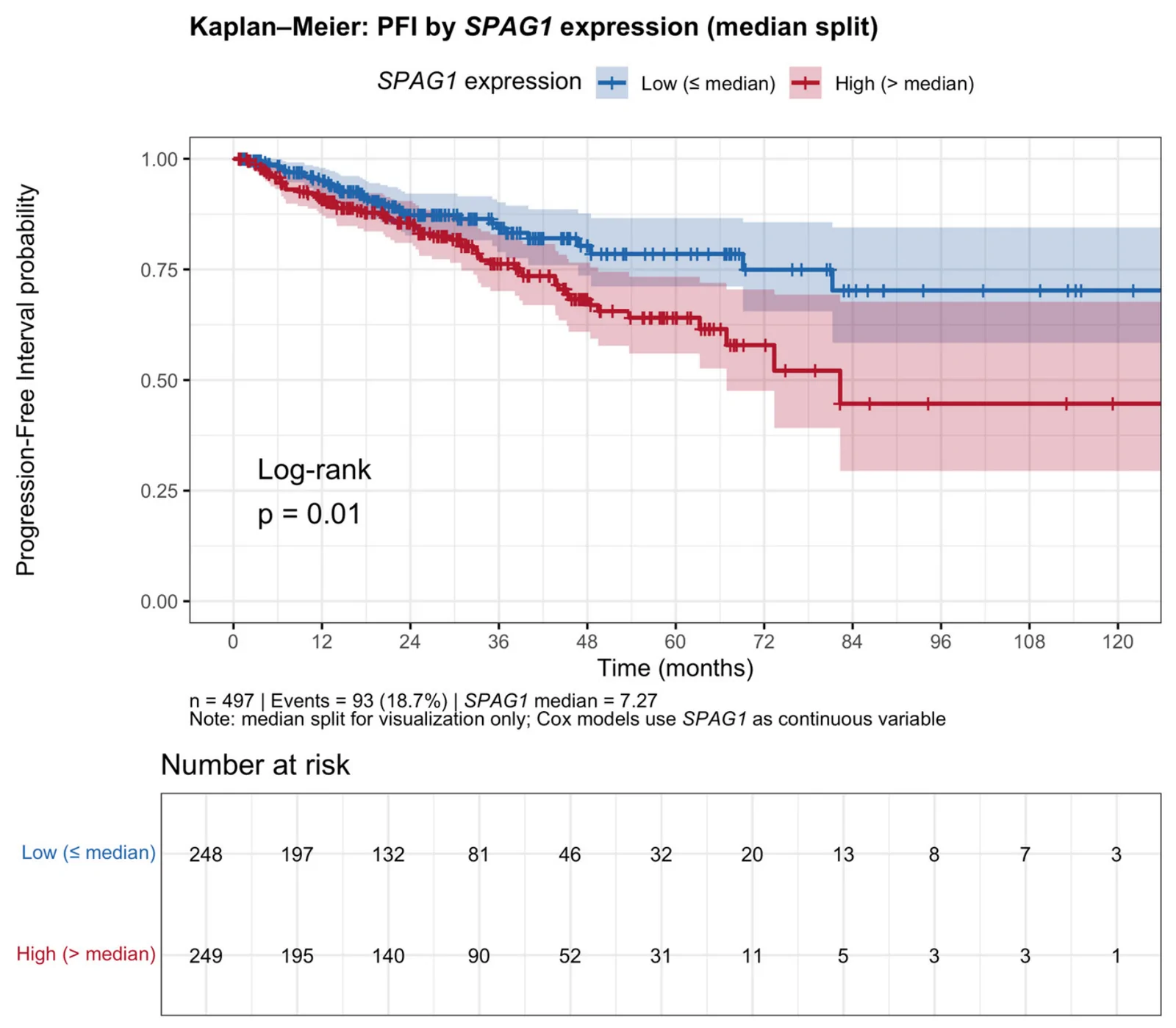
Kaplan–Meier analysis of progression-free interval by *SPAG1* expression. Patients were stratified by median *SPAG1* expression for visualisation only; Cox models used *SPAG1* as a continuous variable. Significance was assessed by the log-rank test. Shaded areas represent 95% confidence intervals; tick marks indicate censored observations. *n* = 497; events = 93.

In the univariable Cox regression analysis (*n* = 497; 93 PFI events), higher *SPAG1* expression was associated with a shorter PFI (hazard ratio [HR], 1.42; 95% CI, 1.12–1.81; *p* = 0.004). Additionally, the Kaplan–Meier analysis using median dichotomisation confirmed worse PFI in the high-expression group (log-rank *p* = 0.011) (Figure 1). After adjusting for the pathological T stage, nodal status, and Gleason grade (*n* = 420 complete cases; 84 PFI events), the *SPAG1* association was attenuated to nonsignificant (adjusted HR, 1.10; 95% CI, 0.82–1.47; *p* = 0.515), and a high Gleason grade and T3/T4 stage remained the strongest independent predictors. This attenuation indicated that the prognostic signal of *SPAG1* is largely encoded within the established clinicopathological stage and does not represent an independent survival effect. The proportional hazards assumption was satisfied for *SPAG1* in both unadjusted and adjusted models with nonsignificant Schoenfeld residual tests (unadjusted *SPAG1*: *p* = 0.43; adjusted *SPAG1*: *p* = 0.21; global: *p* = 0.57).

In an exploratory analysis stratified by nodal status, *SPAG1* expression was not associated with the PFI within node-positive (N1) tumours (*n* = 79, 22 events; univariable HR, 0.96; 95% CI, 0.58–1.60; *p* = 0.887; log-rank *p* = 0.606), whereas an association was present within node-negative (N0) tumours (*n* = 345, 62 events; HR, 1.54; 95% CI, 1.10–2.15; *p* = 0.012; log-rank *p* = 0.029). Both analyses were univariable, and given the limited size of the node-positive subgroup, these stratified estimates should be interpreted with caution (Supplementary Table S1).

### 3.3. SPAG1 Expression Independently Predicted Lymph Node Metastasis

In the complete-case cohort comprising 420 patients (79 patients with N1; 341 patients with N0), N1 rates increased monotonically across *SPAG1* expression quartiles (from 7.6% in Q1 to 39.0% in Q4, indicating a five-fold gradient) (Table 2). In the univariable logistic regression analysis, *SPAG1* was strongly associated with nodal metastasis (OR, 2.86; 95% CI, 2.06–4.08; *p* = 1.5 × 10^−9^). After adjusting for the pathological T stage and Gleason grade, *SPAG1* remained an independent predictor (adjusted OR, 2.14; 95% CI, 1.50–3.13; *p* = 4.8 × 10^−5^); additionally, the T3/T4 stage and high Gleason grade retained significance (Table 3). The wide CI for the T stage reflected the small number of T2 cases among patients with N1 tumours (three of 79 patients) (Table 1). The clinical plus *SPAG1* model showed substantially lower AIC than the clinical baseline (314.4 versus 331.1), an improvement of 16.7 AIC units (likelihood ratio test *p* = 1.55 × 10^−5^), confirming the incremental contribution of *SPAG1* to model fit. The dose–response relationship across quartiles was significant (per-quartile OR, 1.83; 95% CI, 1.39–2.46; *p* = 2.55 × 10^−5^), and AIC values were comparable across continuous, quartile, and trend parameterisations (Supplementary Table S2).

**Figure 2:**
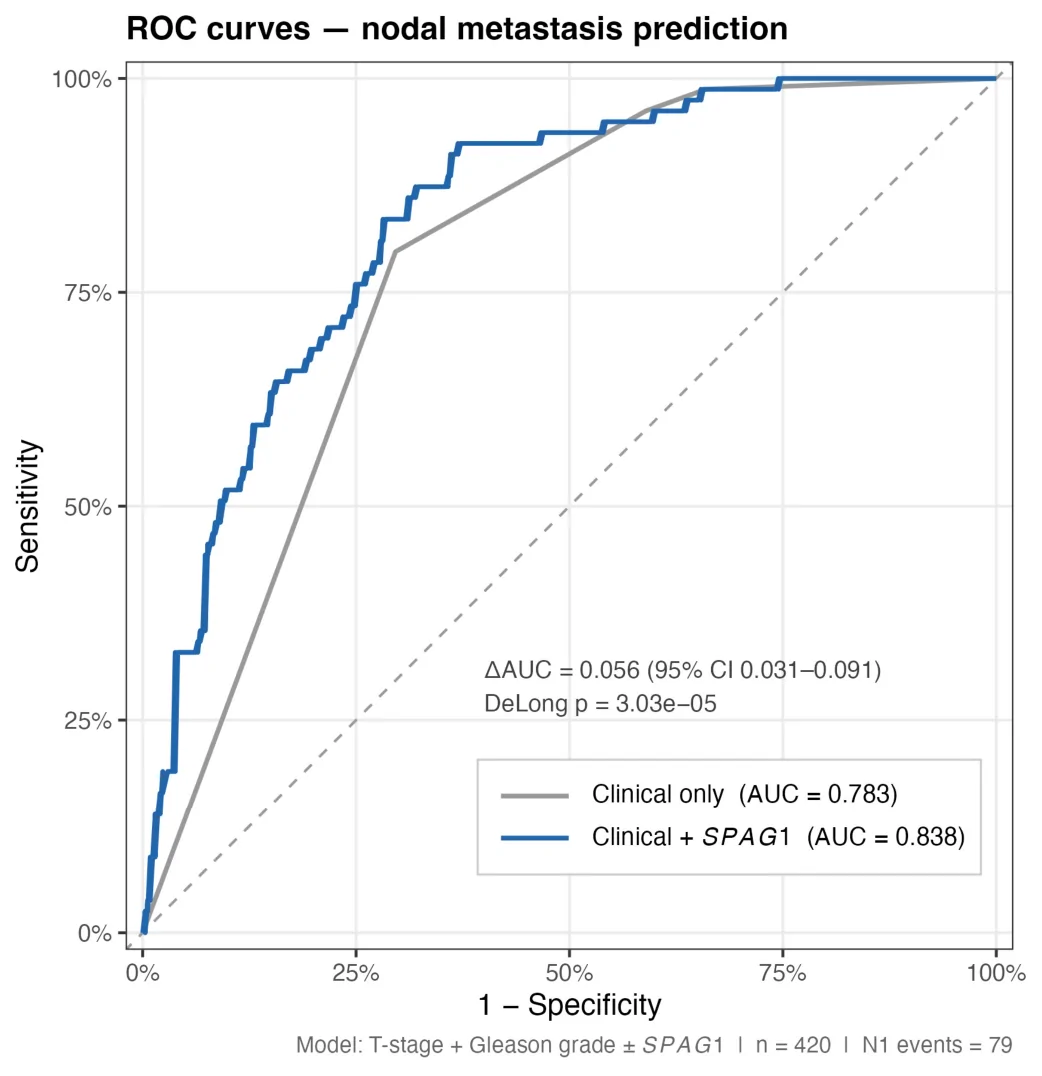
Receiver-operating characteristic curves for prediction of pathological N1 status. The clinical baseline model (T stage + Gleason grade) is compared with the primary model incorporating *SPAG1*. Incremental discrimination was assessed by the paired DeLong test. The diagonal dashed line represents chance. *n* = 420; N1 events = 79.

Adding *SPAG1* to clinical variables significantly improved discrimination, as indicated by the AUC increasing from 0.783 to 0.838 (ΔAUC, 0.056; paired DeLong *p* = 3.03 × 10^−5^; bootstrap 95% CI, 0.031–0.091) (Figure 2). Bootstrap optimism correction (2000 resamples) confirmed minimal overfitting (optimism-corrected AUC, 0.836; optimism, 0.003; calibration slope, 0.942; Hosmer–Lemeshow *p* = 0.672) (Supplementary Figure S3). The *SPAG1* OR was robust across both sensitivity analyses. Reclassification of patients with missing N as node-negative did not change the OR (OR, 2.14; 95% CI, 1.52–3.07); however, additional adjustment for the tissue source site changed the OR by 2.3% (OR, 2.09; 95% CI, 1.45–3.02); this model had borderline events-per-variable [EPV = 9.9], further supporting selection of the more parsimonious primary model (Supplementary Tables S3A and S3B). *SPAG1* expression showed no significant correlation with immune infiltration, stromal content, or tumour purity (Supplementary Table S4).

### 3.4. SPAG1 Transcriptional Programmes Were Concordant with the Molecular Signature of Nodal Metastasis

**Figure 3:**
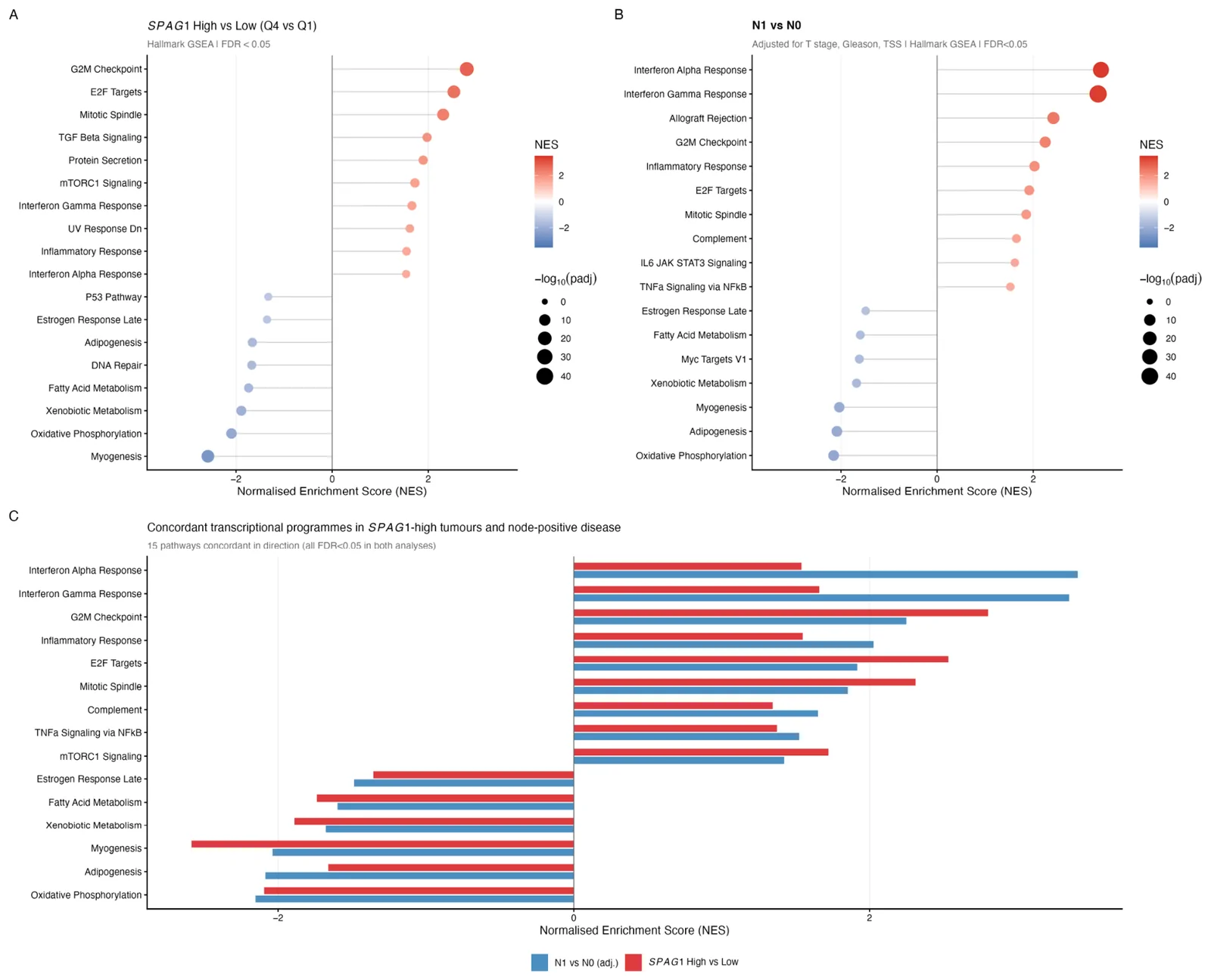
Hallmark gene set enrichment analysis of the *SPAG1*-associated and nodal metastasis transcriptional programmes. (**A**) Enriched Hallmark pathways (FDR < 0.05) comparing *SPAG1* expression extremes (Q4 vs. Q1). (**B**) Enriched Hallmark pathways in the N1 vs. N0 analysis adjusted for T stage, Gleason grade, and tissue source site. In (**A**,**B**), dot colour encodes the normalised enrichment score (NES) and dot size encodes −log_10_(FDR). (**C**) NES values for the 15 pathways significant in both analyses; all 15 were directionally concordant.

A Hallmark GSEA comparing *SPAG1* expression extremes (Q4 vs. Q1, *n* ≈ 125 per group) identified 21 significantly enriched pathways (FDR < 0.05; Supplementary Table S5). The dominant enriched programmes included cell-cycle pathways G2M checkpoint (normalised enrichment score NES, 2.80), E2F targets (NES, 2.53), and Mitotic Spindle (NES, 2.31) alongside immune–inflammatory pathways including interferon-γ response (NES, 1.66), Inflammatory Response (NES, 1.55), and TNFα Signalling via NFκB (NES, 1.37) and signalling pathways including mTORC1 signalling (NES, 1.72) and TGF-β signalling (NES, 1.98). Strong negative enrichment was observed for myogenesis (NES, −2.59), oxidative phosphorylation (NES, −2.10), and Xenobiotic Metabolism (NES, −1.89), reflecting suppression of differentiation and metabolic programmes in *SPAG1*-high tumours (Figure 3A; Supplementary Table S5).

A differential expression analysis that compared N1 and N0 tumours and adjusted for the T stage, Gleason grade, and tissue source site identified 31 differentially expressed genes at FDR < 0.05, with *SPAG1* itself significantly upregulated in N1 tumours (logFC 0.479; padj = 0.013) (Supplementary Table S6A). The adjusted N1 transcriptional signature was dominated by interferon response and inflammatory signalling pathways, alongside upregulation of proliferative programmes including G2M checkpoint and E2F targets (Figure 3B; Supplementary Table S6B). Of the 18 pathways significantly enriched in the adjusted N1 analysis and the 21 in the *SPAG1* analysis, 15 overlapped; all 15 were directionally concordant (Figure 3C; Supplementary Table S6C). This complete directional concordance indicated that high *SPAG1* expression is associated with the same transcriptional state that characterises node-positive disease after accounting for clinical confounders.

Repeating the *SPAG1* high-versus-low GSEA within each nodal stratum identified 28 and 21 significantly enriched Hallmark pathways (FDR < 0.05) in N0 and N1 tumours, respectively (Supplementary Table S7). The proliferative and cell-cycle programme was shared and directionally concordant across strata: G2M checkpoint (N0 NES, 2.33; N1 NES, 2.11), E2F targets, and Mitotic Spindle were enriched in high-*SPAG1* tumours in both N0 and N1, as were mTORC1 signalling and the suppression of myogenesis and oxidative phosphorylation. A notable difference between strata was in the immune–inflammatory pathways: the interferon-γ response and related immune signatures were enriched in high-*SPAG1* node-negative tumours (interferon-γ response N0 NES, 2.16; FDR = 2.3 × 10^−9^) but not in node-positive tumours, where the corresponding enrichment scores approximated or fell below the null value (interferon-γ response N1 NES, 0.71; FDR = 0.99). Thus, the proliferative core of the *SPAG1*-associated programme, together with suppression of myogenesis and oxidative phosphorylation, was preserved irrespective of nodal status, whereas its immune–inflammatory component was largely confined to node-negative tumours. Full results for all 50 Hallmark pathways in both strata are provided in Supplementary Table S7.

## 4. Discussion

In this focused analysis of a cohort from TCGA-PRAD, *SPAG1* expression in primary tumours was independently associated with pathological lymph node metastasis after adjustment for the T stage and Gleason grade (adjusted OR, 2.14; 95% CI, 1.50–3.13; *p* = 4.8 × 10^−5^) and provided incremental discrimination beyond clinical staging (ΔAUC, 0.056; bootstrap-corrected AUC, 0.836). The high-*SPAG1* transcriptional state in primary tumours was concordant with the adjusted molecular signature of node-positive disease. In the same cohort, however, *SPAG1* did not retain prognostic value for the PFI after adjusting for clinicopathological stage, indicating that its association with outcome was largely captured by clinicopathological stage and grade variables and did not represent an independent survival driver.

Our findings build on the prior identification of *SPAG1* as a candidate marker of the nodal status in TCGA-PRAD by Shamsara and Shamsara [10] and add several elements that strengthen the evidence base for prioritising this gene. First, we formally quantified the incremental discriminative value of *SPAG1* beyond a clinical baseline using a paired AUC comparison with bootstrap CIs (ΔAUC, 0.056; 95% CI, 0.031–0.091) and confirmed minimal overfitting using bootstrap optimism correction (optimism-corrected AUC, 0.836), thus providing estimates that are interpretable in the context of contemporary biomarker development. Second, we demonstrated robustness across sensitivity analyses by reclassifying patients with missing nodal status information and adjusting for the tissue source site. Third, we characterised the *SPAG1*-associated transcriptional programme and demonstrated complete directional concordance with the adjusted molecular signature of nodal metastasis across all 15 overlapping Hallmark pathways. Additional analyses related to the primary model, including discrimination and internal validation (Supplementary Table S8), a comparison of SPAG1 expression between patients included in and excluded from the primary complete-case analysis (Supplementary Table S9), and subgroup-specific associations between *SPAG1* expression and pathological N1 status (Supplementary Table S10), are provided in the Supplementary Materials.

Attenuation of the association between *SPAG1* and survival with multivariable adjustment merits explicit interpretation. Although the univariable analysis showed that patients with high *SPAG1* expression had a shorter PFI, this effect was largely accounted for by the pathological T stage and Gleason grade. Therefore, we interpreted *SPAG1* as a molecular correlate of features already encoded within established clinicopathological variables rather than as an independent prognostic biomarker. In the stratified analysis, the univariable *SPAG1*–PFI association was present in node-negative but not node-positive tumours, although the latter subgroup was small (Supplementary Table S1). This interpretation is consistent with our observation that *SPAG1* expression tracks both the pathological T stage and Gleason grade (Supplementary Figure S2B,C). The independent association between *SPAG1* and pathological nodal metastasis in surgical specimens after adjusting for the T stage and Gleason grade established statistical rationales for prioritising *SPAG1* as a candidate for further biomarker development. If these findings are confirmed in independent cohorts, then the most clinically relevant application would be the prediction of nodal involvement at the point of treatment planning; in this setting, improved stratification could refine patient selection for extended pelvic lymph node dissection and complement molecular imaging approaches such as PSMA-PET/CT, particularly when imaging access is limited or findings are equivocal [2]. The present analysis used RNA-seq data from resected primary tumours; however, translation to a preoperative or biopsy-based setting would require the demonstration of *SPAG1* expression in diagnostic biopsy material reflecting the same biology observed in this study. This necessary next step was not performed in this study, however.

Transcriptional concordance between *SPAG1* expression extremes and the adjusted molecular signature of node-positive disease (15 of 15 overlapping pathways were directionally concordant) suggests that *SPAG1* expression captures, at least in part, a transcriptional state that is biologically coherent with nodal spread. The *SPAG1*-associated transcriptional programme was characterised by activation of cell-cycle, immune–inflammatory, and signalling pathways, accompanied by suppression of differentiation and metabolic programmes. Stratified analysis further indicated that the proliferative core of this programme, exemplified by G2M checkpoint enrichment, together with suppression of myogenesis and oxidative phosphorylation, was present in both node-negative and node-positive tumours, whereas the immune–inflammatory component, including the interferon-γ response, was largely confined to node-negative disease; given the smaller number of node-positive tumours, these stratum-specific immune differences should be regarded as exploratory. The persistence of these enrichment patterns within both nodal strata suggests that these components of the *SPAG1* programme are not merely a reflection of nodal status.

The biological functions previously attributed to SPAG1 provide plausible context for these transcriptional findings, although they do not establish a causal mechanism in prostate cancer. In pancreatic cancer cells, SPAG1 colocalised and co-immunoprecipitated with tubulin, while *SPAG1* knockdown impaired transwell migration and wound closure, supporting a possible relationship between SPAG1, tubulin and cellular motility [6]. *SPAG1* knockdown reduced cell proliferation and colony-forming ability in breast cancer cells [8] and in acute myeloid leukaemia cells, induced S-phase accumulation and apoptosis and reduced MEK/ERK phosphorylation and *SMC3* expression [7]. SPAG1 has also been implicated in meiotic spindle organisation and meiotic progression in mouse oocytes [18]. Collectively, these observations are biologically consistent with the enrichment of G2M checkpoint, E2F targets and Mitotic Spindle programmes in *SPAG1*-high prostate tumours. They raise the possibility that *SPAG1* expression identifies a proliferative and cytoskeleton-associated tumour state associated with disease progression and dissemination. Nevertheless, the present data do not demonstrate that *SPAG1* directly activates these pathways or drives lymph node metastasis, and *SPAG1* may instead represent a marker of a broader aggressive transcriptional programme. Functional studies in prostate cancer models will be required to distinguish these possibilities.

This study had several limitations. First, all analyses were performed in a single cohort (TCGA-PRAD) using bulk RNA-seq of resected radical prostatectomy specimens. External validation in independent cohorts was not feasible; among publicly available prostate cancer gene expression datasets with clinical annotation, the available cohorts (GSE21032, GSE54460, GSE70769, GSE70768) either lacked pathological N stage data or contained insufficient N1 events for adequately powered logistic regression. Replication in cohorts with sufficient nodal annotation, and evaluation of *SPAG1* in diagnostic biopsy material by RNA-based assay or immunohistochemistry, are both required before clinical application can be considered. Second, our analyses were observational; functional studies are required to determine whether *SPAG1* has a mechanistic role in nodal dissemination or is a passenger marker. Third, the median follow-up was relatively short (2.16 years; IQR, 1.16–3.73 years), which may have limited the ability to detect long-term survival differences independent of established clinicopathological stage variables.

## 5. Conclusions

*SPAG1* expression is independently associated with pathological lymph node metastasis in prostate cancer and provides modest, but statistically robust, incremental discrimination beyond clinical staging variables. The prognostic association of *SPAG1* was captured by the established stage and grade, and its potential clinical value, pending biopsy-based validation in independent cohorts, may lie in prediction of nodal involvement rather than independent prognostication after radical prostatectomy.

## Figures and Tables

**Table 1:** The clinical and pathological characteristics of the TCGA-PRAD analysis cohort by nodal status (*n* = 497).

Characteristic	N0 (*n* = 345)	N1 (*n* = 79)	Missing *n* (*n* = 73)	*p*-Value ^a^
Age at diagnosis (years), median (IQR)	62 (57–66)	63 (57–68)	59 (54–66)	0.715
PSA at sample collection (ng/mL), median (IQR)	0.10 (0.03–0.11)	0.10 (0.03–0.60)	0.04 (0.03–0.10)	0.187
Pathological T stage, *n* (%)				7.0 × 10^−10^
T2	140 (40.6%)	3 (3.8%)	44 (60.3%)	
T3/T4	201 (58.3%)	76 (96.2%)	26 (35.6%)	
Unknown	4 (1.2%)	0 (0.0%)	3 (4.1%)	
Gleason grade group, *n* (%)				6.1 × 10^−13^
Gleason ≤ 7 (Low/Intermediate)	218 (63.2%)	14 (17.7%)	60 (82.2%)	
Gleason ≥ 8 (High)	127 (36.8%)	65 (82.3%)	13 (17.8%)	
Surgical margin, *n* (%)				9.0 × 10^−9^
Negative	240 (69.6%)	27 (34.2%)	48 (65.8%)	
Positive	89 (25.8%)	46 (58.2%)	17 (23.3%)	
Biochemical recurrence, *n* (%)				0.085
Yes	39 (11.3%)	15 (19.0%)	4 (5.5%)	
No	266 (77.1%)	54 (68.4%)	51 (69.9%)	
Vital status, *n* (%)				0.378
Alive	339 (98.3%)	76 (96.2%)	72 (98.6%)	
Deceased	6 (1.7%)	3 (3.8%)	1 (1.4%)	
Progression-free interval (months), median (IQR)	26.9 (15.6–44.9)	23.9 (11.5–41.0)	27.3 (12.5–41.6)	0.145
PFI events, *n* (%)	62 (18.0%)	22 (27.8%)	9 (12.3%)	
*SPAG1* expression (log_2_ RSEM), median (IQR)	7.22 (6.74–7.67)	7.85 (7.39–8.38)	6.93 (6.57–7.39)	6.3 × 10^−11^

^a^ *p*-values compare N0 vs. N1 groups: Wilcoxon rank-sum for continuous variables; chi-squared or Fisher exact (where appropriate) for categorical variables. NX patients excluded from comparisons. Abbreviations: IQR, interquartile range; PFI, progression-free interval; PRAD, prostate adenocarcinoma; PSA, prostate-specific antigen; RSEM, RNA-seq by Expectation Maximisation; *SPAG1*, sperm-associated antigen 1; TCGA, The Cancer Genome Atlas.

**Table 2:** Pathological N1 status by *SPAG1* expression quartile in the complete-case cohort (*n* = 420).

*SPAG1* Quartile	*n*	N0, *n*	N1, *n*	N1 Rate (%)
Q1 (lowest)	105	97	8	7.6
Q2	105	96	9	8.6
Q3	105	84	21	20.0
Q4 (highest)	105	64	41	39.0

Quartiles defined within the *n* = 420 complete-case cohort (after exclusion of 73 NX patients and 4 patients with missing T stage). Trend across quartiles: *p* = 2.55 × 10^−5^.

**Table 3:** Adjusted logistic regression model for pathological N1 status (primary model, *n* = 420).

Variable	Reference	Adjusted OR	95% CI	*p*-Value
T stage T3/T4	T2	8.59	2.98–36.40	4.9 × 10^−4^
Gleason grade ≥ 8 (High)	Gleason ≤ 7	4.10	2.18–8.15	2.5 × 10^−5^
*SPAG1* expression (per log_2_ RSEM unit)	—	2.14	1.50–3.13	4.8 × 10^−5^

Logistic regression model with N1 status as the outcome and T stage, Gleason grade, and *SPAG1* expression (continuous, per log_2_ RSEM unit) as predictors. The wide confidence interval for T stage reflects the small number of T2 cases with N1 disease (3 of 79). Abbreviations: CI, confidence interval; OR, odds ratio; RSEM, RNA-seq by Expectation Maximisation; *SPAG1*, sperm-associated antigen 1.

## Data Availability

The datasets analysed in this study are publicly available. Clinical, survival, and RNA-sequencing data for TCGA-PRAD were obtained from the UCSC Xena platform (https://xenabrowser.net/datapages/) (accessed on 17 February 2026). The analysis code is openly available on GitHub (https://github.com/Ebtihal-abh/SPAG1-lymph-node-metastasis-TCGA-PRAD) (accessed on 2 June 2026) and archived on Zenodo (https://doi.org/10.5281/zenodo.20513977).

## Supplementary Materials

The following supporting information can be downloaded at https://www.mdpi.com/article/10.3390/genes17080875/s1. Figure S1: Sample selection flow diagram; Figure S2: *SPAG1* expression across clinicopathological features; Figure S3: Calibration of the primary logistic regression model; Table S1: Univariable association between *SPAG1* expression (continuous) and the progression-free interval within nodal strata; Table S2: Logistic regression model selection and parameterisation; Table S3A: Robustness of the *SPAG1*–N1 association across sensitivity analyses; Table S3B: Events-per-variable (EPV) for primary and TSS-adjusted models; Table S4: Spearman correlations between *SPAG1* expression and tumour microenvironmental composition in TCGA-PRAD; Table S5: Hallmark fgsea results—*SPAG1* Q4 vs. Q1; Table S6A: Differentially expressed genes (FDR < 0.05): N1 vs. N0, adjusted for T stage, Gleason grade, and tissue source site; Table S6B: Hallmark pathways enriched at FDR < 0.05: N1 vs. N0 (adjusted); Table S6C: Pathway-level concordance between adjusted N1 vs. N0 and *SPAG1* Q4 vs. Q1 analyses; Table S7: *SPAG* high-versus-low Hallmark gene set enrichment analysis within nodal strata (median split of *SPAG1* expression within each stratum); Table S8: Discrimination and internal validation of the primary logistic regression model; Table S9: Comparison of *SPAG1* expression between patients included in (n = 420) and excluded from (n = 77) the primary complete-case analysis; Table S10: Association between *SPAG1* expression and pathological N1 status within clinicopathological subgroups.
