# Supplementary material for: SPAG1 Expression as a Candidate Predictor of Pathological Lymph Node Metastasis in Prostate Cancer: A Transcriptomic Analysis of The Cancer Genome Atlas Prostate Adenocarcinoma Cohort
Source: Genes (Basel). 2026 Jul 28;17(8):875. doi: 10.3390/genes17080875 (PMC13511855; doi:10.3390/genes17080875)
Supplement: Supplementary file 1 [file genes-17-00875-s001.zip › Supplementary Figure S1.pdf]

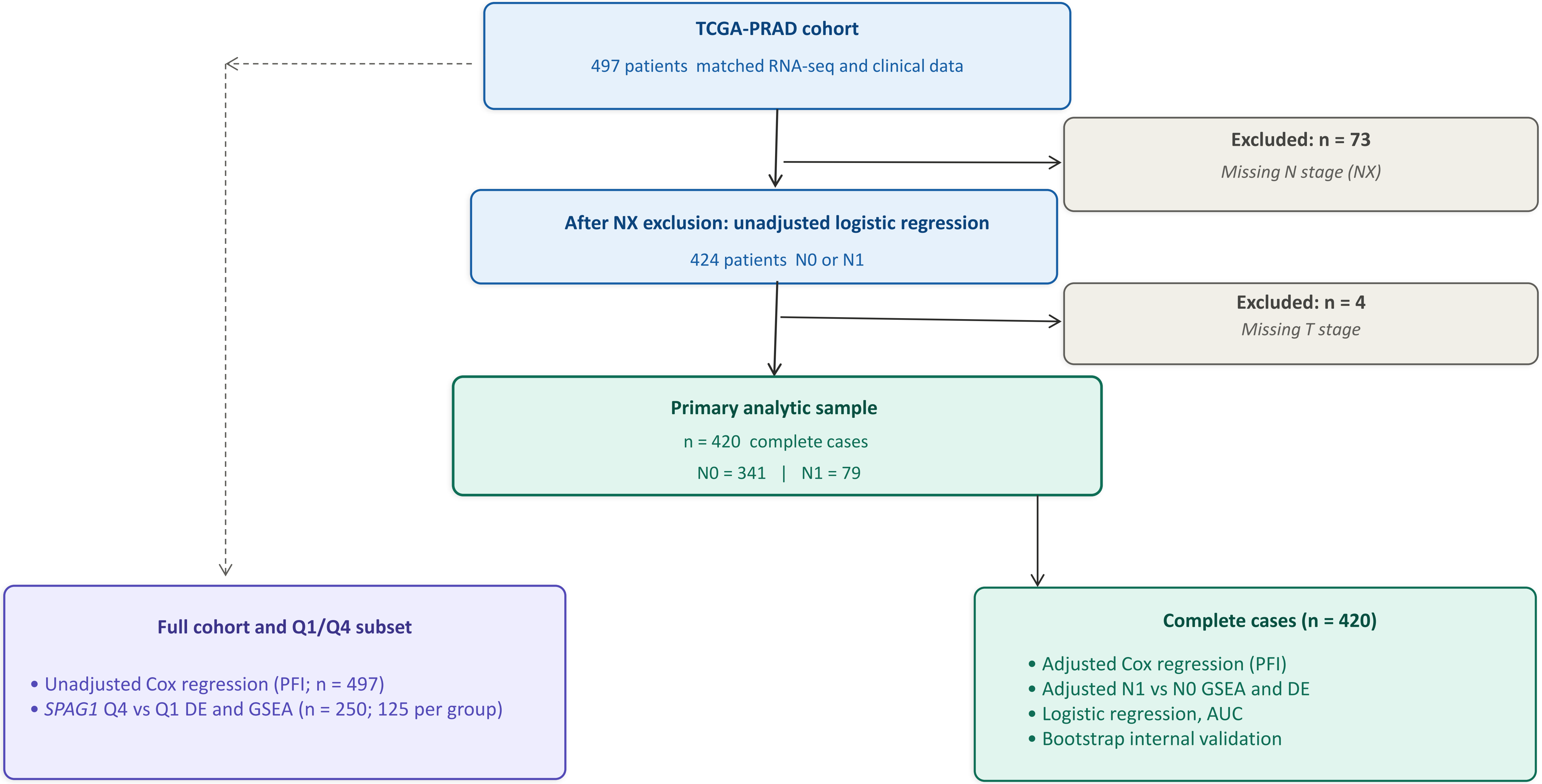

Sensitivity analyses: NX reclassified as N0 (n = 490); logistic models additionally adjusted for tissue source site. Adjusted DE and GSEA models include T stage, Gleason grade, and tissue source site as covariates.
